# Epidemiological Trends of COVID‐19 Infection and Symptom Incidence in China Following the Adjustment of Zero‐COVID Policy: A Prospective, Community‐Based Cohort Study

**DOI:** 10.1155/tbed/5590977

**Published:** 2026-05-05

**Authors:** Zhigao Chen, Zhen Zhang, Yeen Huang, Zhanwei Du, Ruohan Chen, Nixuan Chen, Tingting Liu, Yanpeng Cheng, Honglin Wang, Huawei Xiong, Lixia Song, Yanfen Ye, Yan Lu, Ziquan Lv, Tingting Cao, Yingying Li, Bin Zhu, Xuan Zou, Jianhua Lu, Shenying Fang, Benjamin J. Cowling

**Affiliations:** ^1^ Shenzhen Center for Disease Control and Prevention, Shenzhen, Guangdong, China, szcdc.net; ^2^ School of Public Health and Emergency Management, Southern University of Science and Technology, Shenzhen, Guangdong, China, sustc.edu.cn; ^3^ WHO Collaborating Centre for Infectious Disease Epidemiology and Control, School of Public Health, Li Ka Shing Faculty of Medicine, The University of Hong Kong, Hong Kong Special Administrative Region, Pokfulam, China, hku.hk; ^4^ Laboratory of Data Discovery for Health Limited, Hong Kong Science and Technology Park, New Territories, Hong Kong Special Administrative Region, Hong Kong, China

**Keywords:** China, cohort study, COVID-19, epidemiology, SARS-CoV-2

## Abstract

This study tracked the postadjustment infection rates and symptom trends within community settings. Conducted from May 2023 to May 2024 across four districts in Shenzhen, involving 3246 participants, over 80% had received at least one vaccine dose. Postpolicy adjustment witnessed three significant infection surges. Among the cohort, 63.7% reported only one Coronavirus disease 2019 (COVID‐19) infection, 30.3% two infections, and 1.7% three infections, with 4.3% remaining uninfected throughout the follow‐up. Older adults and those with lower IgG levels had increased reinfection risk. Notably, 3.8% (95% CI: 3.1–4.6) reported long COVID, with higher susceptibility in those with underlying conditions (adjusted odds ratio [AOR] = 3.73, 95% CI: 2.20–6.34) and reduced incidence among fully vaccinated individuals (AOR = 0.57, 95% CI: 0.36–0.90). The policy change led to widespread exposure, shifting from first infections to reinfections. These insights underscore the need for ongoing research to inform future pandemic responses.

## 1. Introduction

Between 2020 and 2021, the Coronavirus disease 2019 (COVID‐19) pandemic resulted in an estimated 14.83–18.20 million excess deaths worldwide [1, 2]. Beyond fatalities, prolonged symptoms (long COVID) continue to impose a substantial disease burden on 30%–50% of survivors [3, 4], indicating the pandemic’s lingering impact on public health [5].

COVID‐19 trends have shown regional variations, heavily influenced by the diverse containment strategies of different countries [6–8]. Initially, countries relied on various nonpharmaceutical interventions (NPIs) like school closures and mobility restrictions [9, 10]. However, the effectiveness of these interventions varied [11], leading to multiple infection waves despite the introduction of vaccines in 2021 [12–15]. Global vaccine acceptance and uptake rates showed significant disparities [16], and new variants prompted surges in cases despite adjustments in NPIs [17]. By 2022 and 2023, the emergence of Omicron variants led to a shift towards relaxing restrictions, especially in high‐income economies, while low‐income economies maintained stringent measures [18].

Distinct from most countries, China maintained the zero‐COVID strategy for almost 3 years, effectively controlling virus transmission early on and significantly reducing the reproduction number (*R*‐value) [18–21]. China’s transition away from this policy in December 2022 led to immediate nationwide infection peaks. Notably, strict COVID‐19 measures also affected the epidemic trends of other seasonal respiratory diseases [22, 23], highlighting the necessity of continued virus trend monitoring in the post‐COVID era [24–26].

The distinct epidemic trends across regions, influenced by dominant strains, population immunity, and demographic factors, have led to unique patterns of COVID‐19 infection and symptom occurrence [27, 28]. Prior infections have shown varying degrees of protective effects against reinfections, especially with Omicron subvariants [29]. Studies also highlight a significant proportion of asymptomatic infections [30], particularly with Omicron subvariants [31]. and an increasing focus on the multifaceted impacts of long COVID [32, 33].

The evolving nature of COVID‐19 presents significant differences from its initial spread in 2020 [34–37], raising critical questions about the virus’s mutating impact on the general and particularly vulnerable populations. Current evidence, mainly from hospital‐based or specific cohorts, lacks the generalizability needed for comprehensive public health strategies. China’s strict zero‐COVID approach provides a unique dataset to observe Omicron infection and symptom trends across uninfected and reinfected populations.

This study, utilizing a prospective community‐based cohort in China, aims to investigate COVID‐19 infection rates, symptom occurrences, and long COVID incidences postpolicy adjustment. By analyzing reinfections and symptoms during periods dominated by new viral strains, the study seeks to offer insights into infection trends and risk factors in the general population. This will address existing knowledge gaps and inform future public health policies and resource allocation.

## 2. Methods

### 2.1. Study Design and Participants

**Figure 1:**
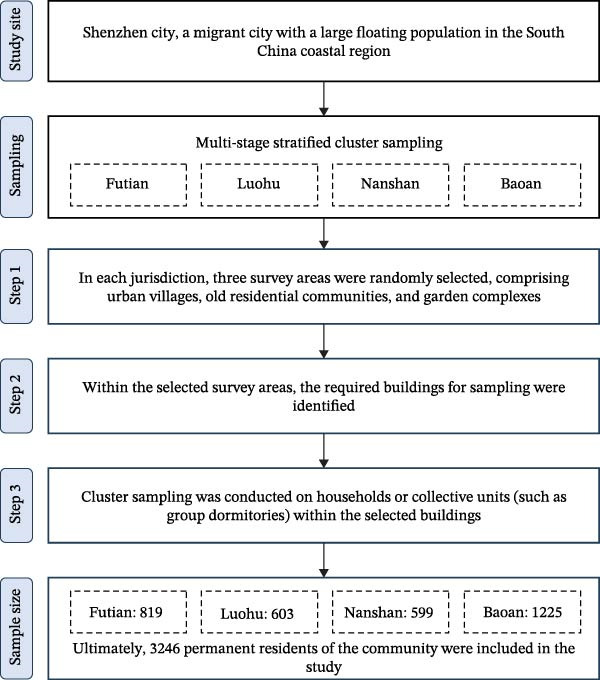
Sample Collection. This study is a 1‐year prospective community‐based cohort study conducted from May 2023 to May 2024 across four districts (study sites) in Shenzhen. Participants were recruited using a multistage stratified cluster sampling method at each study site, resulting in a total of 3246 permanent residents from the community.

This 1‐year prospective, community‐based cohort study (Shenzhen community population COVID‐19 study cohort) spanned from May 2023 to May 2024 in Shenzhen, a major migrant city in South China with a substantial floating population. The study covered Futian, Luohu, Nanshan, and Baoan districts, selected based on geographical location, economic status, and population size. A multistage stratified cluster sampling was employed: initially, three communities, including urban villages, old residential areas, and garden communities, were randomly selected from each district. Subsequently, specific buildings within these communities were identified for sampling, followed by cluster sampling of households or collective units (e.g., dormitories). A total of 3246 community residents were ultimately included in the study (see Figure 1).

The study was approved by the Shenzhen Center for Disease Control and Prevention Ethics Committee (Ethics ID: 2023027A). All participants provided informed consent, with adults signing for themselves and guardians signing for minors under 18 years of age.

### 2.2. Data Collection: An Integrated Intelligent System

In collaboration with Sinosoft Company Limited, a comprehensive digital system for data collection and quality control was developed, enhancing the convenience and accuracy of cohort data collection. This system, operational on web and WeChat mini‐program platforms, integrated survey, nucleic acid sampling data, and backend data analysis functionalities. Survey data were entered and managed electronically, with immediate logic and data completeness checks by surveyors, allowing data managers to promptly identify and correct errors.

### 2.3. Baseline Survey

Preliminary surveys in two Futian district communities ensured the feasibility of the study protocol and questionnaire data usability. All surveyors, samplers, and laboratory personnel underwent standardized training to ensure data consistency. The baseline survey, conducted from May 19 to June 1, 2023, involved face‐to‐face questionnaire interviews by trained personnel at community health centers, covering demographics, past COVID‐19 infections, acute symptoms postinfection, and long COVID symptoms (for detailed measurement methods, refer to the Methods section of Supporting Information). Blood samples for baseline antibody (IgG and IgM) testing and throat swab samples for nucleic acid testing were collected from all participants. Telephone follow‐ups verified potentially biased past COVID‐19 infection reports and questionnaire data.

### 2.4. Follow‐Up Survey

To prospectively monitor participants for initial or subsequent COVID‐19 infections and the occurrence of acute symptoms, a 1‐year follow‐up survey was conducted from May 2023 to May 2024. The frequency of follow‐up was adjusted based on whether it was a COVID‐19 epidemic period (defined as when the proportion of influenza‐like cases in sentinel hospitals exceeds 2.5% for three consecutive days and the detection rate of COVID‐19 in fever clinics exceeds 10.0%) to bi‐weekly (during nonepidemic periods) or weekly (during epidemic periods). The follow‐up included nucleic acid testing (similar to the baseline survey; if participants were unable to attend in person, they could conduct an antigen test and report the results to the researchers) and electronic follow‐up questionnaires (only required for those who tested positive for the virus), which detailed the occurrence of acute symptoms postinfection (including the nine symptoms identified in the baseline survey).

### 2.5. Data Quality Control

This study implemented several measures for data quality control, including the use of an integrated intelligent system (see Section 2.2), presurveys, and telephone follow‐ups. Before the official survey, presurveys were conducted in two communities in the Futian district to ensure the feasibility of the study process and the usability of the questionnaire. After the baseline survey, for participants who self‐reported never having been infected with COVID‐19, telephone follow‐ups were conducted to reverify past infection information and questionnaire data that may be subject to recall bias.

### 2.6. Statistical Analysis

Statistical analyses were performed with IBM SPSS Statistics Version 26.0 (IBM Corp, Armonk, NY, USA) and R Version 4.2.2 (R Foundation for Statistical Computing, Vienna, Austria), considering *p*‐value <0.05 as statistically significant. The study’s high follow‐up rate (average 87.1%) allowed for comprehensive data analysis.

To address potential recall bias and misclassification, participants’ self‐reported infection history was adjusted using baseline IgG and IgM levels (see Figure S1 for correction details). Descriptive statistics summarized baseline demographics, COVID‐19 infection history, symptom occurrence, and long COVID prevalence, using means (SDs) or medians (IQRs) for quantitative data and frequencies (percentages) for categorical data. Infection rates and symptom distribution over time were analyzed using histograms and frequency distributions. Univariate and multivariate Cox regression models identified risk factors for a second COVID‐19 infection. Survival time was defined as the period between the first and second infections, with uncensored data for those not reinfected by the last follow‐up. Results included hazard ratios (HRs), adjusted HR, and confidence intervals (CIs). Symptom incidence differences between first and second infections were analyzed using chi‐square tests for both independent and paired samples. Univariate and multivariate logistic regression models were employed to explore factors associated with the occurrence of acute symptoms postinfection and long COVID. The analyses focused on the presence of any acute or long COVID symptoms, reporting odds ratios (ORs), adjusted OR (AOR), and CI.

## 3. Results

Table 1 shows that among the 3246 participants, 56.2% were female, with an average age (standard deviation) of 39.7 (17.5) years. The highest proportion of participants was in the 36–60 age group (48.5%), with half of the participants’ baseline IgG and IgM levels falling within the second quartile (P25–P75). Over 80.0% had received at least one dose of a COVID‐19 vaccine, and 10.8% reported having one or more chronic or other underlying diseases.

**Table 1:** Baseline characteristics of participants, *N* = 3246.

Variables	*N* (%)
Total	3246 (100.0)
Gender	
Male	1423 (43.8)
Female	1823 (56.2)
Age (years)	
<18	455 (14.0)
18–35	801 (24.7)
36–60	1573 (48.5)
>60	417 (12.8)
Mean (SD)	39.7 (17.5)
Education level	
Junior high school or below	890 (27.4)
Senior high school or vocational school	731 (22.5)
College, university or above	1625 (50.1)
Occupational categories	
Manual laborers	1281 (39.5)
Nonmanual laborers	822 (25.3)
Unemployed or retired individuals	818 (25.2)
Others or freelancers	325 (10.0)
BMI (kg/m^2^), mean (SD)	22.30 (3.98)
Classification of baseline antibody levels	
IgG (mg/mL)	
First quartile (<P25)	800 (25.0)
Second quartile (P25–P75)	1599 (50.0)
Third quartile (>P75)	800 (25.0)
Missing	47
Median (IQR)	87.07 (101.78)
IgM (mg/mL)	
First quartile (<P25)	794 (24.8)
Second quartile (P25–P75)	1604 (50.2)
Third quartile (>P75)	801 (25.0)
Missing	47
Median (IQR)	0.20 (0.23)
Number of doses of COVID‐19 vaccine administered	
0	621 (19.1)
1 or 2	625 (19.3)
3 or 4	2000 (61.6)
Presence of chronic or other underlying diseases	
No	2897 (89.2)
Yes	349 (10.8)

*Note:* P25, 25th percentile; P75, 75th percentile.

Abbreviations: BMI, body mass index; IQR, interquartile range; SD, standard deviation.

**Figure 2:**
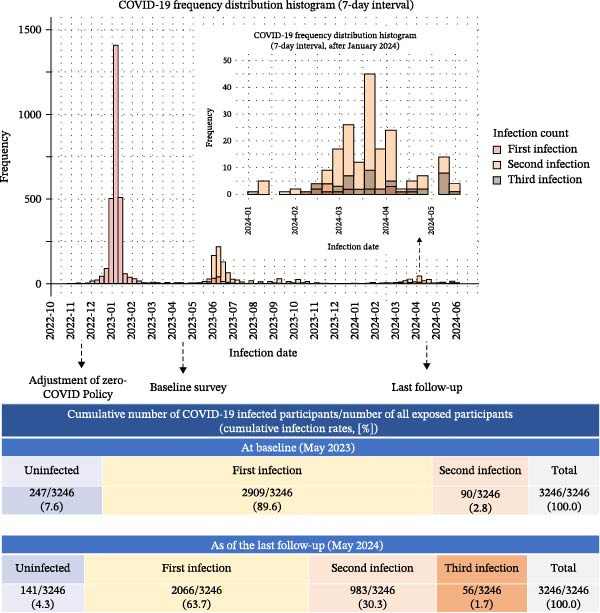
Temporal distribution of first, second, and third COVID‐19 infections among participants infected. Since the adjustment of the zero‐COVID policy to the last follow‐up of this study, there have been three major peaks in infections, the first between December 2022 and January 2023, the second between May and June 2023, and the third between March and April 2024. Up to the most recent follow‐up, 1.7% of the participants had been infected with COVID‐19 three times, 30.3% had been infected twice, 63.7% once, and 4.3% remained uninfected.

Figures 2 and S2 depict changes in COVID‐19 infection rates following the zero‐COVID policy adjustment. Three infection peaks occurred after the policy adjustment up to the last follow‐up in May 2024 (Figure 2), the first between December 2022 and January 2023, the second between May and June 2023, and the third between March and April 2024. After adjusting for self‐reported past infections and baseline antibody levels, 2909 (89.6%) and 90 (2.8%) individuals experienced their first and second infection, and 247 individuals (7.6%) remained uninfected. By the last follow‐up, 56 (1.7%), 983 (30.3%), and 2066 (63.7%) participants had cumulatively experienced their third, second, and first COVID‐19 infections, respectively, with 141 individuals (4.3%) still uninfected.

In the first wave (Dec 2022–Jan 2023), all infections were first infections (100.0%) (Figure S3). By the second wave (May–Jun 2023), second infections dominated (673 cases, 84.2%), with fewer first infections (125 cases, 15.6%) and two third infections (0.3%). In the third wave (Mar–Apr 2024), most documented infections were second infections (153 cases, 78.1%), third infections increased (33 cases, 16.8%), and first infections were now infrequent (10 cases, 5.1%). By the end of the third wave in April 2024, only 4.3% of the cohort had not reported at least one infection. This Kaplan–Meier curve (Figure S4) shows the survival probability from the first to the second and from the second to the third COVID‐19 infections. The survival probability for a second infection decreases more quickly than for a third infection, indicating faster occurrence of second infection. Within a year of infection, the risk of second infection was estimated to be 24.4%, and within a year after the second infection the risk of a third infection, was estimated to be 4.9%.

We used Cox regression models to explore potential risk factors for a second COVID‐19 infection among those already infected once. Univariate (Table S1) and multivariate (Table 2) analyses identified age and baseline IgG levels as main risk factors. Compared to participants under 18, those aged 18–35 (adjusted HR = 1.61, 95% CI: 1.02–2.56), aged 36–60 (adjusted HR = 1.98, 95% CI: 1.27–3.09) and over 60 (adjusted HR = 1.91, 95% CI: 1.28–2.86) had a higher risk of reinfection. Participants with IgG levels in the third quartile (adjusted HR = 0.76, 95% CI: 0.61–0.93) had a lower risk of reinfection compared to those in the first quartile.

**Table 2:** Factors associated with the risk of second infection in individuals with a first COVID‐19 infection: multivariate Cox regression analysis, *N* = 2909.

Variables	Incidence of second infection (%)	Risk of second infection^a^		
		Multivariate Cox regression analysis		
		Adjusted HR	95% CI	*p*‐Value
Total	30.6	–	–	–
Gender				
Male	30.9	1.00	–	–
Female	30.4	0.94	0.82–1.07	0.338
Age (years)				
<18	18.8	1.00	–	–
18–35	28.9	1.61	1.02–2.56	0.042
36–60	34.3	1.98	1.27–3.09	0.002
>60	32.4	1.91	1.28–2.86	0.002
Education level				
Junior high school or below	25.9	1.00	–	–
Senior high school or vocational school	32.2	0.99	0.79–1.23	0.918
College, university or above	32.5	0.98	0.78–1.22	0.839
Occupational categories				
Manual laborers	32.5	1.00	–	–
Nonmanual laborers	32.4	1.01	0.84–1.21	0.926
Unemployed or retired individuals	24.3	0.93	0.71–1.20	0.569
Others or freelancers	34.5	1.06	0.85–1.33	0.601
BMI (kg/m^2^)	–	1.00	0.98–1.02	0.859
Classification of baseline antibody levels				
IgG				
First quartile (<P25)	36.3	1.00	–	–
Second quartile (P25–P75)	31.2	0.87	0.74–1.03	0.099
Third quartile (>P75)	26.2	0.76	0.61–0.93	0.007
IgM				
First quartile (<P25)	34.4	1.00	–	–
Second quartile (P25–P75)	31.1	0.94	0.80–1.11	0.476
Third quartile (>P75)	27.6	0.83	0.68–1.02	0.069
Number of doses of COVID‐19 vaccine administered				
0	30.1	1.00	–	–
1 or 2	24.6	1.17	0.89–1.53	0.251
3 or 4	32.7	1.05	0.88–1.25	0.598
Presence of chronic or other underlying diseases				
No	30.3	1.00	–	–
Yes	33.6	1.06	0.85–1.33	0.610

*Note:* P25, 25th percentile; P75, 75th percentile.

Abbreviations: BMI, body mass index; CIs, confidence intervals; HRs, hazard ratios.

^a^Among participants with a history of one COVID‐19 infection at baseline, the occurrence of a second infection was considered the dependent variable. Survival time was defined as the interval between the first infection and the second infection (or until the final follow‐up in this study). If no second infection occurred by the last follow‐up, the survival time was defined as the interval between the first infection and the last follow‐up. A Cox regression model was employed to investigate risk factors associated with the occurrence of a second COVID‐19 infection, with all factors satisfying the proportional hazards assumption.

To assess the impact of increased COVID‐19 infection occurrences on acute symptom onset, symptom incidence rates were compared between different participants (Table 3) and within the same participants (Table 4) for first and second infections. Compared across different participants, those reinfected had a significantly lower proportion of experiencing one or more symptoms compared to those infected once (55.9% vs. 88.5%, *p*  < 0.001), with fever showing the most significant drop in occurrence (25.1% vs. 73.5%, *p*  < 0.001). Intraparticipant comparison yielded similar results.

**Table 3:** Comparison of symptom incidence rates between different participants for first and second COVID‐19 infection, *N* = 2925.

Symptoms	First infection, *N* = 1948^a^	Second infection, *N* = 977^b^	*χ* ^2^	*p*‐Value
	*n* (%)	*n* (%)		
Presence of any symptom^c^	—	—	398.29	<0.001
No	224 (11.5)	431 (44.1)	—	—
Yes	1724 (88.5)	546 (55.9)	—	—
Fever	—	—	622.53	<0.001
No	517 (26.5)	732 (74.9)	—	—
Yes	1431 (73.5)	245 (25.1)	—	—
Cough/expectoration	—	—	241.80	<0.001
No	725 (37.2)	661 (67.7)	—	—
Yes	1223 (62.8)	316 (32.3)	—	—
Fatigue	—	—	79.90	<0.001
No	855 (43.9)	600 (61.4)	—	—
Yes	1093 (56.1)	377 (38.6)	—	—
Reduced sense of smell/taste	—	—	0.02	0.898
No	1418 (72.8)	709 (72.6)	—	—
Yes	530 (27.2)	268 (27.4)	—	—
Nasal congestion	—	—	89.43	<0.001
No	1133 (58.2)	742 (75.9)	—	—
Yes	815 (41.8)	235 (24.1)	—	—
Runny nose	—	—	44.19	<0.001
No	1192 (61.2)	719 (73.6)	—	—
Yes	756 (38.8)	258 (26.4)	—	—
Sore throat	—	—	435.91	<0.001
No	1012 (52.0)	889 (91.0)	—	—
Yes	936 (48.0)	88 (9.0)	—	—
Conjunctivitis	—	—	28.25	<0.001
No	1882 (96.6)	900 (92.1)	—	—
Yes	66 (3.4)	77 (7.9)	—	—
Diarrhea	—	—	2.60	0.107
No	1821 (93.5)	928 (95.0)	—	—
Yes	127 (6.5)	49 (5.0)	—	—

^a^The first infection refers to participants who had only one COVID‐19 infection by the final follow‐up of this study (2066 participants). Those who did not report symptoms during their first infection were excluded (118 participants with missing data), resulting in a sample size of 1948 for analysis.

^b^The second infection refers to participants who had two COVID‐19 infections by the final follow‐up (983 participants). Those who did not report symptoms during their second infection were excluded (six participants with missing data), resulting in a sample size of 977 for analysis.

^c^Participants were defined as symptomatic if they experienced any of the following symptoms: fever, cough/expectoration, fatigue, reduced sense of smell/taste, nasal congestion, runny nose, sore throat, conjunctivitis, or diarrhea. The duration of symptoms, self‐reported by participants, was categorized as 1–3 days, 4–7 days, 1–2 weeks, or 2–4 weeks and classified as “Yes” for analysis.

**Table 4:** Comparison of symptom incidence rates within the same participants for first and second COVID‐19 infection, 887/894.

Symptoms	First infection, *N* = 887^a^	Second infection, *N* = 894^a^	*χ* ^2b^	*p*‐Value
	*n* (%)	*n* (%)		
Presence of any symptom^c^	—	—	269.55	<0.001
No	89 (10.0)	428 (47.9)	—	—
Yes	798 (90.0)	466 (52.1)	—	—
Fever	—	—	402.65	<0.001
No	227 (25.6)	699 (78.2)	—	—
Yes	660 (74.4)	195 (21.8)	—	—
Cough/expectoration	—	—	140.59	<0.001
No	309 (34.8)	634 (70.9)	—	—
Yes	578 (65.2)	260 (29.1)	—	—
Fatigue	—	—	169.91	<0.001
No	363 (40.9)	569 (63.6)	—	—
Yes	524 (59.1)	325 (36.4)	—	—
Reduced sense of smell/taste	—	—	0.66	0.416
No	660 (74.4)	650 (72.7)	—	—
Yes	227 (25.6)	244 (27.3)	—	—
Nasal congestion	—	—	123.61	<0.001
No	493 (55.6)	709 (79.3)	—	—
Yes	394 (44.4)	185 (20.7)	—	—
Runny nose	—	—	67.76	<0.001
No	527 (59.4)	691 (77.3)	—	—
Yes	360 (40.6)	203 (22.7)	—	—
Sore throat	—	—	154.45	<0.001
No	397 (44.8)	866 (96.9)	—	—
Yes	490 (55.2)	28 (3.1)	—	—
Conjunctivitis	—	—	10.37	0.001
No	849 (95.7)	823 (92.1)	—	—
Yes	38 (4.3)	71 (7.9)	—	—
Diarrhea	—	—	5.55	0.019
No	823 (92.8)	853 (95.4)	—	—
Yes	64 (7.2)	41 (4.6)	—	—

^a^By the final follow‐up of this study, 983 participants had experienced two COVID‐19 infections. After excluding those who did not report symptoms during the first infection (96 missing data) or the second infection (89 missing data), the sample sizes available for analysis were 887 and 894, respectively.

^b^The McNemar test was used to compare the distribution of symptom occurrence between the first and second COVID‐19 infections within the same participants.

^c^Participants were defined as symptomatic if they experienced any of the following symptoms: fever, cough/expectoration, fatigue, reduced sense of smell/taste, nasal congestion, runny nose, sore throat, conjunctivitis, or diarrhea. Symptom duration, self‐reported by participants, was categorized as 1–3 days, 4–7 days, 1–2 weeks, or 2–4 weeks and classified as “Yes” for analysis.

We used logistic regression models to explore potential factors influencing acute symptom occurrence post‐first or second COVID‐19 infections. For acute symptoms post‐first infection, univariate (Table S2) and multivariate (Table S3) analyses showed that infections occurring after March 2023 (during XBB.1.9.1/XBB.1.16/EG.5.1/JN.1 dominance) were associated with a lower risk of symptom occurrence (AOR = 0.09, 95% CI: 0.06–0.13). Participants with a higher BMI had a higher risk of developing symptoms (AOR = 1.06, 95% CI: 1.01–1.10).

For second infections, factors influencing acute symptom occurrence differed from those for first infections (Tables S2 and S3). Participants symptomatic during their first infection had a higher risk of symptoms upon reinfection (AOR = 2.01, 95% CI: 1.27–3.16). Participants over the age of 60 had a lower risk of reinfection symptoms than participants under 18 (AOR = 0.32, 95% CI: 0.15–0.72). Notably, as with the first infections, reporting chronic diseases was a risk factor for symptoms in second infections (AOR = 1.66, 95% CI: 1.05–2.62).

Among participants with one or more past infections, 3.8% (95% CI 3.1–4.6) reported long COVID symptoms (Table 5). Univariate (Table S4) and multivariate (Table 5) analyses indicated that women were more likely to develop long COVID postinfection (AOR = 2.21, 95% CI: 1.41–3.47), as were those with chronic or underlying diseases (AOR = 3.73, 95% CI: 2.20–6.34). Vaccination with three or four doses reduced the risk of long COVID (AOR = 0.57, 95% CI: 0.36–0.90).

**Table 5:** Factors associated with the risk of Long COVID occurrence in COVID‐19 infected individuals: multivariate logistic regression analysis, *N* = 2868^**a**^.

Variables	Long COVID (lasting >3 months)^a^			
	Presence of any long COVID (%)	Risk of any long COVID occurrence (Multivariate logistic regression analysis)^b^		
		Adjusted OR	95% CI	*p*‐Value
Total	3.8	–	–	–
Gender				
Male	2.3	1.00	–	–
Female	4.9	2.21	1.41–3.47	0.001
Age (years)				
<18	0.8	1.00	–	–
18–35	2.3	2.26	0.49–10.31	0.295
36–60	3.6	3.18	0.73–13.85	0.123
>60	3.0	1.75	0.42–7.26	0.439
Education level				
Junior high school or below	2.2	1.00	–	–
Senior high school or vocational school	2.7	0.95	0.47–1.89	0.874
College, university or above	3.2	1.64	0.86–3.13	0.136
Occupational categories				
Manual laborers	3.3	1.00	–	–
Nonmanual laborers	2.7	0.95	0.57–1.59	0.845
Unemployed or retired individuals	2.2	1.01	0.51–2.03	0.973
Others or freelancers	3.0	0.89	0.46–1.73	0.729
BMI (kg/m^2^)	–	0.96	0.91–1.02	0.201
Number of doses of COVID‐19 vaccine administered				
0	4.0	1.00	–	–
1 or 2	1.9	0.61	0.27–1.36	0.224
3 or 4	2.7	0.57	0.36–0.90	0.016
Presence of chronic or other underlying diseases				
No	2.5	1.00	–	–
Yes	5.5	3.73	2.20–6.34	<0.001

Abbreviations: BMI, body mass index; CIs, confidence intervals; ORs, Odds ratios.

^a^There were 131 cases with missing data on “Long COVID” symptoms.

^b^Participants were defined as having “Long COVID” if they experienced any of the following symptoms with a duration >3 months: cough, fatigue, reduced sense of smell/taste, myalgia, diarrhea, dizziness/headache, sleep disturbances, cognitive impairments, mood disorders, chest tightness/breathing difficulties, loss of appetite, rash, or hair loss.

^c^Among participants who had one or two COVID‐19 infections at baseline, the occurrence of any “Long COVID” (symptom duration >3 months) was considered the dependent variable. A logistic regression model was used to investigate the risk factors associated with the development of “Long COVID.”

## 4. Discussion

The lack of general population data in previous studies may introduce biases in estimating COVID‐19 trends. Our community‐based cohort study in Shenzhen, China, during the monitoring period from Dec 2022 to May 2024, reveals three infection peaks (Dec 2022–Jan 2023, May–Jun 2023, and Mar–Apr 2024), with 63.7% infected once, 30.3% twice, and 1.7% three times; 4.3% remained uninfected. The infection patterns across the three waves showed a shift from exclusively first infections in the first wave to second infections dominating in the second and third waves, alongside a gradual increase in third infections and a decline in first infections. Older individuals and those with lower antibody levels faced higher reinfection risks. Notably, acute symptoms decreased in subsequent infections, with underlying diseases posing a significant risk for both acute symptoms and long COVID, whereas vaccination offered protective effects.

Initially, infection rate changes were closely linked to regional containment policies and the virus’s transmission capability. Studies comparing early pandemic responses and epidemiological outcomes in locations like Singapore, Korea, Japan, Taiwan, and Hong Kong highlighted the effectiveness of rapid control measures [15]. However, most locations reported higher peaks during the Delta variant dominance [12]. A study encompassing 190 countries estimated that ~43.9% of the global population had been infected at least once by November 2021 [38]. Our study aligns with these findings, indicating a significant infection peak in December 2022 once the zero‐COVID policies were relaxed, similar to estimates suggesting nearly 97% of Mainland China’s population was infected with the Omicron variant around December 23, 2022 [39]. These results emphasize the efficacy of swift and strict containment strategies in delaying nationwide outbreaks during a pandemic’s early stages [19, 40].

We found that reinfection risk was related to antibody levels, sociodemographic characteristics, etc. A study in Shanghai showed a self‐reported reinfection rate of 9.72%, with higher risks among severely infected initial patients [41]. Our study’s higher reinfection rate (30.3%) might be due to differences in study follow‐up duration and social measures. We found higher reinfection risks in individuals aged over 18, with higher IgG levels associated with lower risks. Notably, our findings diverged from previous studies, possibly due to differences in participant selection [28, 29, 41–44], as our study focused on community residents.

The symptomatology of secondary infections during the initial COVID‐19 variant waves varied, with some reports indicating similar [27], more severe [45], or milder [46] symptoms than the first infection. After the emergence of Omicron and its variants, the proportion of asymptomatic infections seemed to increase [31, 47]. Our study compared symptom incidences of first and second Omicron infections among different individuals and within the same individuals, finding an increase in asymptomatic proportions with subsequent infections. Differences in asymptomatic frequency estimates across studies may result from incomplete symptom assessments, insufficient follow‐up, and misclassification, among other factors [28, 41, 48–52].

Estimates of long COVID prevalence vary significantly across studies, with recent meta‐analyses reporting rates of 30%–45% [3, 53]. Our community‐based study found a 3.8% prevalence of long COVID among survivors, aligning with some previous findings [54, 55]. Multivariate analyses identified being female and having underlying diseases as risk factors for long COVID, consistent with prior research [55, 56]. Interestingly, our findings suggest vaccination (three or four doses) provides protection against long COVID, similar to other studies suggesting vaccination reduces prolonged postinfection symptoms [49].

## 5. Strengths and Limitations

This study’s strengths include being one of the first prospective cohort studies based on the community population to observe COVID‐19 trends, providing strong causal inference and generalizability compared to retrospective and hospital‐based studies. The use of digital systems, presurveys, and telephone follow‐ups for data quality control minimizes the risk of information and recall biases. Selecting community residents and conducting follow‐ups at nearby health centers ensured a high follow‐up rate (87.1%), allowing for timely identification of new infections and symptoms.

However, limitations include reliance on self‐reported data, potentially overlooking other dimensions like environmental and genetic factors, and the inability to identify the specific SARS‐CoV‐2 strains in positive cases, possibly introducing misclassification bias. Adjustments for baseline COVID‐19 status aimed to reduce recall bias but could not entirely eliminate misclassification potential. Further research is needed to accurately assess long COVID occurrences and elucidate its pathophysiological mechanisms.

## 6. Conclusion

Following the zero‐COVID policy adjustment, our findings show that most individuals have contracted the virus at least once, with older people and those with lower antibody levels more prone to reinfections. Across the three infection waves, the infection pattern shifted from exclusively first infections in the first wave to a dominance of second infections in the subsequent waves, along with a gradual increase in third infections and a decline in first infections. Infection intervals also progressively lengthened, with significant differences observed between intervals. Despite a higher asymptomatic rate in subsequent infections, those with pre‐existing conditions are at increased risk for acute symptoms and long COVID, whereas vaccination effectively reduces these risks. Our study offers crucial epidemiological insights into COVID‐19’s evolving trends within the general population, underlining the need for further research to decode these trends’ mechanisms and enhance pandemic preparedness.

## Acknowledgments

The authors extend their sincere gratitude to all participants who contributed to this study. Additionally, heartfelt thanks are extended to all staff members involved in this cohort study for their invaluable efforts.

## Funding

This work was supported by the Special Foundation of Science and Technology Innovation Strategy of Guangdong Province of China (Grant 2020B1111340077).

## Disclosure

The funder of the study had no role in study design, data collection, data analysis, data interpretation, or writing of the report. All authors critically revised the manuscript for important intellectual content and agreed to submit the final version for publication. All authors agree to be accountable for all aspects of the work in ensuring that questions related to the accuracy or integrity of any part of the work are appropriately investigated and resolved. All authors had full access to all the data in the study and had final responsibility for the decision to submit for publication.

## Conflicts of Interest

Benjamin J. Cowling has consulted for AstraZeneca, Fosun Pharma, GlaxoSmithKline, Haleon, Moderna, Novavax, Pfizer, Roche, and Sanofi Pasteur. The other authors declare no conflicts of interest.

## Supporting Information

Additional supporting information can be found online in the Supporting Information section.

## Supporting information


**Supporting Information** The Supporting Information provides more detailed information of methods. Figure S1: Process of correction for baseline COVID‐19 infection status, *N* = 3246. Figure S2: Trends in COVID‐19 infection rates. Figure S3: Temporal distribution and proportions of COVID‐19 infections across waves by infection type. Figure S4: Kaplan–Meier survival curves for time to second and third COVID‐19 infections. Table S1: Factors associated with the risk of second infection in individuals with a first COVID‐19 infection, *N* = 2909. Table S2: Factors associated with the risk of symptom occurrence in COVID‐19 infected individuals: univariate logistic regression analysis, *N* = 2066/983. Table S3: Factors associated with the risk of symptom occurrence in COVID‐19 infected individuals: multivariate logistic regression analysis, *N* = 2066/983. Table S4: Factors associated with the risk of Long COVID occurrence in COVID‐19 infected individuals: univariate logistic regression analysis, *N* = 2868.

## Data Availability

These data are not publicly available. Data can be made available upon reasonable request to the corresponding author and with the permission of the Shenzhen Center for Disease Control and Prevention.
