# Supplementary material for: Epidemiological Trends of COVID‐19 Infection and Symptom Incidence in China Following the Adjustment of Zero‐COVID Policy: A Prospective, Community‐Based Cohort Study
Source: Transbound Emerg Dis. 2026 May 5;2026:5590977. doi: 10.1155/tbed/5590977 (PMC13140162; doi:10.1155/tbed/5590977)
Supplement: Supplementary file 1 — Supporting Information The Supporting Information provides more detailed information of methods. Figure S1: Process of correction for baseline COVID‐19 infection status, N = 3246. Figure S2: Trends in COVID‐19 infection rates. Figure S3: Temporal distribution and proportions of COVID‐19 infections across waves by infection type. Figure S4: Kaplan–Meier survival curves for time to second and third COVID‐19 infections. Table S1: Factors associated with the risk of second infection in individuals with a first COVID‐19 infection, N = 2909. Table S2: Factors associated with the risk of symptom occurrence in COVID‐19 infected individuals: univariate logistic regression analysis, N = 2066/983. Table S3: Factors associated with the risk of symptom occurrence in COVID‐19 infected individuals: multivariate logistic regression analysis, N = 2066/983. Table S4: Factors associated with the risk of Long COVID occurrence in COVID‐19 infected individuals: univariate logistic regression analysis, N = 2868. [file TBED-2026-5590977-s001.docx]

**Supplementary materials**

**Methods**

**2.4 Baseline survey**

Preliminary surveys in two Futian district communities ensured the feasibility of the study protocol and questionnaire data usability. All surveyors, samplers, and laboratory personnel underwent standardized training to ensure data consistency. The baseline survey, conducted from May 19 to June 1, 2023, involved face-to-face questionnaire interviews by trained personnel at community health centers, covering demographics, past COVID-19 infections, acute symptoms post-infection, and Long COVID symptoms. Blood samples for baseline antibody (IgG and IgM) testing and throat swab samples for nucleic acid testing were collected from all participants. Telephone follow-ups verified potentially biased past COVID-19 infection reports and questionnaire data.

**2.4.1 Demographic characteristics**

The demographic characteristics of the study cohort included gender (male, female), age, levels of education (junior high school or lower, high school or vocational training, college, university or higher), occupational classification (Non-specialized occupations, Technical or vocational occupations, Specialized and higher education occupations, Other occupations, Non-occupied individuals), and Body Mass Index (BMI).^1^ Participants were also asked to report the quantity of COVID-19 vaccine doses administered (0, 1 or 2, 3 or 4 doses) along with the respective timing of each dose. Furthermore, the prevalence of chronic diseases or other underlying medical conditions was recorded, including but not limited to diabetes, hypertension, obesity, chronic obstructive pulmonary disease (COPD), stroke, heart disease, chronic kidney disease, allergic asthma, or a combination of these or other foundational health conditions.

**2.4.2 Previous COVID-19 infection history**

To ascertain the past infection status of participants with regard to COVID-19, the survey employed the item, “Have you contracted COVID-19 or experienced symptoms related to COVID-19 after December 2022?” In cases where participants self-reported previous COVID-19 infections, the survey further inquired about the frequency and timing of these infections through the questions, “How many times have you contracted COVID-19?” and “What were the dates of each infection?”

**2.4.3 Symptoms post-most recent infection**

In accordance with previous related studies^2,3^, participants who self-reported a history of COVID-19 infection were required to detail the acute symptoms experienced following their most recent infection. These encompassed a spectrum of nine symptoms: fever, cough or expectoration, fatigue, reduced sense of smell/taste, nasal congestion, runny nose, sore throat, conjunctivitis, and diarrhea. Additionally, the duration of each symptom was categorized into five levels: asymptomatic, lasting 1-3 days, 4-7 days, 1-2 weeks, and 2-4 weeks. In this study, the occurrence of an acute symptom, such as “fever”, was acknowledged if the self-reported duration was 1-3 days or longer. Furthermore, participants were considered to have exhibited symptoms post-infection if they experienced any one or a combination of these nine symptoms.

**2.4.4 Long COVID symptoms**

According to the World Health Organization’s definition, Long COVID, also known as “Post COVID Condition”, refers to symptoms persisting for at least two months beyond three months following the initial SARS-CoV-2 infection, which cannot be explained by an alternative diagnosis.^4^ In the baseline survey of this study, participants who self-reported a previous COVID-19 infection (with the majority having their initial infection more than three months prior to the survey) were asked to report the presence of Long COVID symptoms. These encompassed thirteen symptoms: cough, fatigue, reduced sense of smell or taste, myalgia, diarrhea, dizziness or headaches, sleep disturbances (insomnia, hypersomnia, or hyposomnia), cognitive impairment (reduced memory, lack of concentration), mood disorders (low mood, loss of interest or pleasure, anxiety, depression), chest tightness or breathing difficulties, loss of appetite, rash, and hair loss. The duration of each symptom was categorized into five levels: none, lasting 0-1 month, 1-2 months, 2-3 months, and more than 3 months.^4,5^ In this study, a specific Long COVID symptom, such as “cough”, was considered present if its self-reported duration was “>3 months”. Additionally, the occurrence of any one or a combination of these Long COVID symptoms post-infection was taken as indicative of Long COVID manifestation in the participant.

**2.4.5 Baseline antibody testing**

Previous systematic reviews have indicated that antibody testing can be used to identify past SARS-CoV-2 infections, particularly with a combined IgG/IgM test showing a total sensitivity of 96.0% (95% CI: 90.6-98.3) after the third week of symptom onset.^6^ To ascertain participants’ past COVID-19 infection status and current antibody levels, all subjects underwent SARS-CoV-2 IgG and IgM antibody testing at baseline. All recruited participants visited their local community health service centers at predetermined times set by the research team. There, healthcare personnel at these centers collected blood samples using non-anticoagulant vacutainer tubes (5ml for individuals aged 18 and above, and 3ml for those under 18 years). Following blood collection, serum separation was immediately conducted by the Center for Disease Control and Prevention (CDC) associated with each research site, and the SARS-CoV-2 IgG and IgM antibody testing for each participant was completed within 7 days.

**2.4.6 COVID-19 nucleic acid testing**

To determine the baseline COVID-19 infection status of participants, all subjects were required to undergo nucleic acid testing for SARS-CoV-2. The healthcare personnel at community health service centers collected oropharyngeal swab samples from the participants using non-inactivating sample collection tubes containing 3-4 ml of sampling fluid. Following sample collection, the CDC associated with each research site completed the nucleic acid testing for each participant within 24 hours.

**References**

1. Wang L, Zhou B, Zhao Z, et al. Body-mass index and obesity in urban and rural China: findings from consecutive nationally representative surveys during 2004-18. *Lancet* 2021; **398**(10294): 53-63.

2. Zhang H, Lu Z, Yang F, et al. Symptom profiles and vaccination status for COVID-19 after the adjustment of the dynamic zero-COVID policy in China: An observational study. *J Med Virol* 2023; **95**(7): e28893.

3. Qin S, Li Y, Wang L, Zhao X, Ma X, Gao GF. Assessment of vaccinations and breakthrough infections after adjustment of the dynamic zero-COVID-19 strategy in China: an online survey. *Emerg Microbes Infect* 2023; **12**(2): 2258232.

4. Soriano JB, Murthy S, Marshall JC, Relan P, Diaz JV, Condition WHOCCDWGoP-C-. A clinical case definition of post-COVID-19 condition by a Delphi consensus. *Lancet Infect Dis* 2022; **22**(4): e102-e7.

5. Munblit D, Nicholson T, Akrami A, et al. A core outcome set for post-COVID-19 condition in adults for use in clinical practice and research: an international Delphi consensus study. *Lancet Respir Med* 2022; **10**(7): 715-24.

6. Deeks JJ, Dinnes J, Takwoingi Y, et al. Antibody tests for identification of current and past infection with SARS-CoV-2. *Cochrane Database Syst Rev* 2020; **6**(6): CD013652.


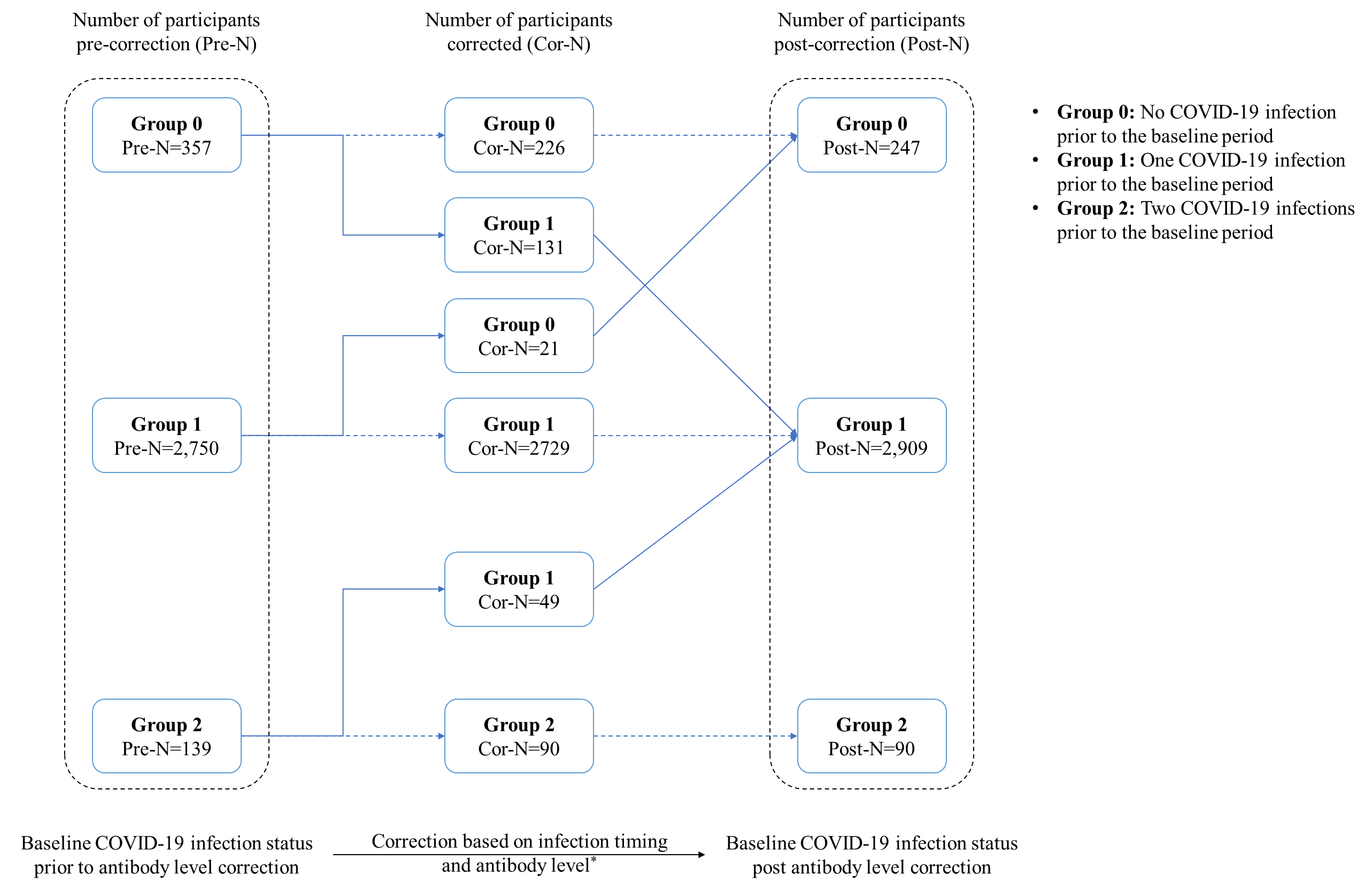


**Supplementary Figure 1. Process of correction for baseline COVID-19 infection status, N=3,246.**

^*^Basic principles for the correction of baseline COVID-19 infection status based on infection timing and antibody levels:

1. Participants who had self-reported no pre-baseline COVID-19 infection, but exhibited antibody levels (IgG and IgM) significantly higher than other uninfected participants, were reclassified as having had one pre-baseline infection.
2. For participants who had elf-reported one pre-baseline COVID-19 infection, a comprehensive assessment was conducted based on infection timing and antibody levels. Among participants with similar infection timing, individuals whose antibody levels (IgG and IgM) were substantially lower than others with one pre-baseline infection, were reclassified as uninfected pre-baseline. Conversely, if their antibody levels were significantly higher, they were reclassified as having had two pre-baseline infections.
3. For participants who had self-reported two pre-baseline COVID-19 infections, a comprehensive assessment based on infection timing and antibody levels was also conducted. Among participants with similar infection timings, individuals whose antibody levels (IgG and IgM) were markedly lower than others with two pre-baseline infections, were reclassified as having had one pre-baseline infection.

**Supplementary Figure 2. Trends in COVID-19 infection rates.** At baseline, 89.6% of the participants had been infected with COVID-19 once, 2.8% had been infected twice, and 7.6% had not yet been infected. The figure illustrates the reinfection rates during the follow-up period among participants with different baseline infection statuses.

**
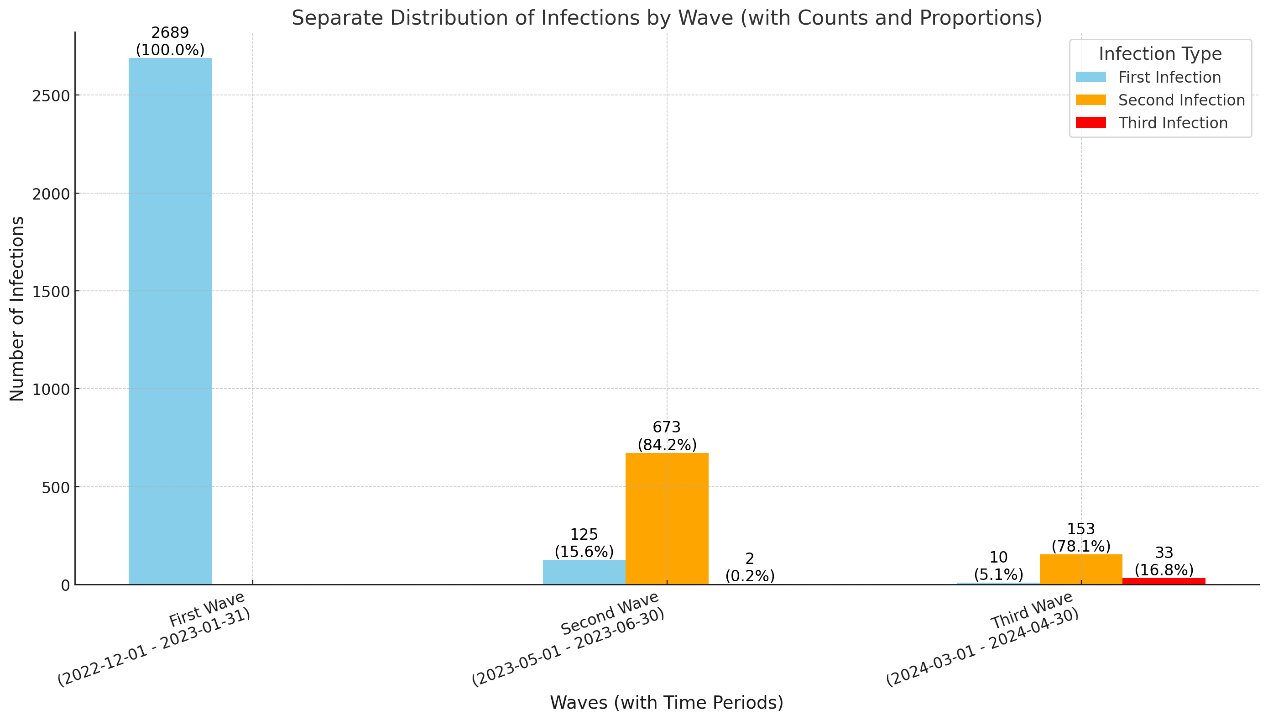
**

**Supplementary Figure 3. Temporal distribution and proportions of COVID-19 infections across waves by infection type**

**
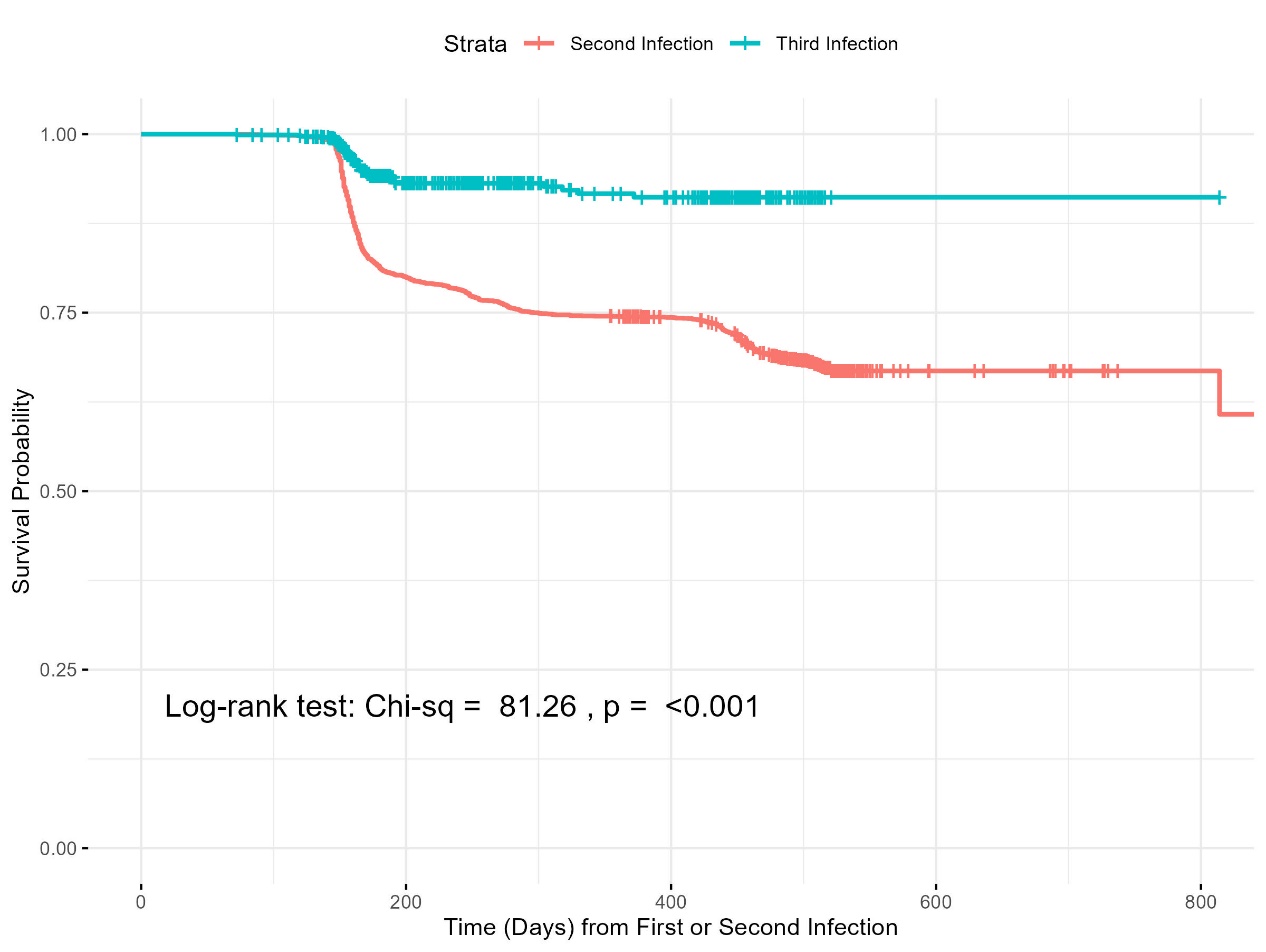
**

**Supplementary Figure 4. Kaplan-Meier survival curves for time to second and third COVID-19 infections.** This Kaplan-Meier curve illustrates the survival probability from the first to the second, and from the second to the third COVID-19 infections. The x-axis represents time (in days) from the first or second infection, and the y-axis shows the probability of remaining free of a second or third infection. A log-rank test shows a significant difference between the curves (Chi-square=81.26, *P*<0.001), indicating that the survival probability for second infection is lower than for third infection. This suggests that individuals in the second infection group experience infection more quickly in the earlier period, leading to a lower survival probability.

**Supplementary Table 1. Factors associated with the risk of second infection in individuals with a first COVID-19 infection, N=2,909.**

| **Variables** | **Incidence of second infection** | **Risk of second infection^a^** | | |
| --- | --- | --- | --- | --- |
|  |  | **Univariate Cox regression analysis** | | |
|  | **%** | **HR** | **95% CI** | ***P-value*** |
| **Total** | 30.6 | - | - | - |
| **Gender** |  |  |  |  |
| Male | 30.9 | 1.00 | - | - |
| Female | 30.4 | 0.93 | 0.82-1.06 | 0.262 |
| **Age (years)** |  |  |  |  |
| <18 | 18.8 | 1.00 | - | - |
| 18-35 | 28.9 | **1.46** | **1.13-1.90** | **0.004** |
| 36-60 | 34.3 | **1.84** | **1.45-2.33** | **<0.001** |
| >60 | 32.4 | **1.79** | **1.35-2.37** | **<0.001** |
| **Education level** |  |  |  |  |
| Junior high school or below | 25.9 | 1.00 | - | - |
| Senior high school or vocational school | 32.2 | **1.25** | **1.03-1.50** | **0.022** |
| College, university or above | 32.5 | **1.19** | **1.01-1.40** | **0.033** |
| **Occupational categories** |  |  |  |  |
| Manual laborers | 32.5 | 1.00 | - | - |
| Non-manual laborers | 32.4 | 0.97 | 0.83-1.14 | 0.725 |
| Unemployed or retired individuals | 24.3 | 0.76 | 0.64-0.91 | 0.002 |
| Others or freelancers | 34.5 | 1.03 | 0.83-1.28 | 0.816 |
| **BMI (kg/m^2^)** | - | **1.02** | **1.01-1.03** | **0.023** |
| **Classification of baseline antibody levels** |  |  |  |  |
| **IgG** |  |  |  |  |
| First quartile (<P25) | 36.3 | 1.00 | - | - |
| Second quartile (P25-P75) | 31.2 | **0.80** | **0.69-0.94** | **0.005** |
| Third quartile (>P75) | 26.2 | **0.70** | **0.58-0.84** | **<0.001** |
| **IgM** |  |  |  |  |
| First quartile (<P25) | 34.4 | 1.00 | - | - |
| Second quartile (P25-P75) | 31.1 | **0.91** | **0.78-1.06** | **0.214** |
| Third quartile (>P75) | 27.6 | **0.81** | **0.67-0.97** | **0.021** |
| **Number of doses of COVID-19 vaccine administered** |  |  |  |  |
| 0 | 30.1 | 1.00 | - | - |
| 1 or 2 | 24.6 | 0.81 | 0.65-1.01 | 0.057 |
| 3 or 4 | 32.7 | 1.07 | 0.90-1.26 | 0.452 |
| **Presence of chronic or other underlying diseases** |  |  |  |  |
| No | 30.3 | 1.00 | - | - |
| Yes | 33.6 | 1.18 | 0.97-1.43 | 0.105 |

Abbreviation: HR, Hazards ratios; CI, confidence intervals; BMI, body mass index; P25, 25th percentile; P75, 75th percentile;

^a^In participants with a history of one COVID-19 infection at baseline, the occurrence of a second infection was used as the dependent variable. The survival time was defined as the interval between the occurrence of the first infection and the time of the second infection until the last follow-up of this study (if no second infection occurred as of the last follow-up, the survival time was defined as the interval between the first infection and the last follow-up). Cox regression model was employed to investigate the factors influencing the risk of second infection with COVID-19. All factors satisfied the proportional hazards assumption.

**Supplementary Table 2. Factors associated with the risk of symptom occurrence in COVID-19 infected individuals: Univariate Logistic regression analysis, N=2,066/983.**

| **Variables** | **First infection, N=2,066** | | | |  | **Second infection, N=983** | | | |
| --- | --- | --- | --- | --- | --- | --- | --- | --- | --- |
|  | **Prevalence of any symptom^a^** | **Risk of any symptom occurrence**  **(Univariate Logistic regression analysis)^b^** | | |  | **Prevalence of any symptom^a^** | **Risk of any symptom occurrence**  **(Univariate Logistic regression analysis)^b^** | | |
|  | **%** | **OR** | **95% CI** | ***P-value*** |  | **%** | **OR** | **95% CI** | ***P-value*** |
| **Total** | 81.4 | - | - | - |  | 55.5 | - | - | - |
| **Timing of the first COVID-19 infection** |  |  |  |  |  |  |  |  |  |
| Before Feb 2023 (BA.5.2/BF.7 predominance period) | 89.6 | 1.00 | - | - |  | 56.6 | 1.00 | - | - |
| After March 2023 (XBB.1.9.1/XBB.1.16/EG.5.1/JN.1 predominance period) | 45.2 | **0.10** | **0.07-0.14** | **<0.001** |  | 39.1 | 0.49 | 0.21-1.15 | 0.493 |
| **Presence of any symptoms during the first infection^a^** |  |  |  |  |  |  |  |  |  |
| No | - | - | - | - |  | 40.4 | 1.00 | - | - |
| Yes | - | - | - | - |  | 57.8 | **2.02** | **1.30-3.15** | **0.002** |
| **Gender** |  |  |  |  |  |  |  |  |  |
| Male | 79.2 | 1.00 | - | - |  | 53.7 | 1.00 | - | - |
| Female | 83.0 | **1.28** | **1.03-1.60** | **0.028** |  | 56.9 | 1.14 | 0.88-1.47 | 0.322 |
| **Age (years)** |  |  |  |  |  |  |  |  |  |
| <18 | 76.4 | 1.00 | - | - |  | 52.4 | 1.00 | - | - |
| 18-35 | 85.0 | **1.75** | **1.24-2.49** | **0.002** |  | 63.1 | 1.55 | 0.94-2.57 | 0.088 |
| 36-60 | 81.0 | 1.32 | 0.98-1.78 | 0.072 |  | 56.6 | 1.18 | 0.74-1.88 | 0.482 |
| >60 | 81.9 | 1.40 | 0.93-2.09 | 0.107 |  | 38.4 | **0.57** | **0.32-0.99** | **0.047** |
| **Education level** |  |  |  |  |  |  |  |  |  |
| Junior high school or below | 76.7 | 1.00 | - | - |  | 49.1 | 1.00 | - | - |
| Senior high school or vocational school | 79.8 | 1.20 | 0.89-1.61 | 0.234 |  | 53.3 | 1.18 | 0.82-1.72 | 0.376 |
| College, university or above | 84.8 | **1.70** | **1.32-2.19** | **<0.001** |  | 59.1 | **1.50** | **1.09-2.06** | **0.013** |
| **Occupational categories** |  |  |  |  |  |  |  |  |  |
| Manual laborers | 81.3 | 1.00 | - | - |  | 54.4 | 1.00 | - | - |
| Non-manual laborers | 84.9 | 1.29 | 0.95-1.74 | 0.101 |  | 63.5 | **1.46** | **1.06-1.99** | **0.019** |
| Unemployed or retired individuals | 78.5 | 0.84 | 0.64-1.09 | 0.193 |  | 44.7 | **0.68** | **0.48-0.96** | **0.028** |
| Others or freelancers | 80.6 | 0.95 | 0.64-1.42 | 0.816 |  | 58.8 | 1.19 | 0.78-1.82 | 0.408 |
| **BMI (kg/m^2^)** | - | **1.03** | **1.01-1.06** | **0.045** |  | - | 0.99 | 0.95-1.02 | 0.476 |
| **Number of doses of COVID-19 vaccine administered** |  |  |  |  |  |  |  |  |  |
| 0 | 82.0 | 1.00 | - | - |  | 55.9 | 1.00 | - | - |
| 1 or 2 | 77.1 | 0.74 | 0.53-1.04 | 0.086 |  | 51.0 | 0.82 | 0.53-1.27 | 0.371 |
| 3 or 4 | 82.6 | 1.05 | 0.78-1.41 | 0.761 |  | 56.5 | 1.02 | 0.74-1.42 | 0.891 |
| **Presence of chronic or other underlying diseases** |  |  |  |  |  |  |  |  |  |
| No | 81.2 | 1.00 | - | - |  | 55.5 | 1.00 | - | - |
| Yes | 82.9 | 1.12 | 0.77-1.63 | 0.548 |  | 56.1 | 1.03 | 0.69-1.52 | 0.892 |

Abbreviation: OR, Odds ratios; CI, confidence intervals; BMI, body mass index;

^a^The presence of any symptom among fever, cough/expectoration, fatigue, reduced sense of smell/taste, nasal congestion, runny nose, sore throat, conjunctivitis, or diarrhea was defined as having symptoms. Participants who self-reported symptom duration in the ranges of 1-3 days, 4-7 days, 1-2 weeks, or 2-4 weeks were classified under the category ‘Yes’.

^b^Among participants who contracted COVID-19 once or twice by the time of the last follow-up, the occurrence of any infection symptom was used as the dependent variable. A Logistic regression models was utilized to explore factors associated with the occurrence of symptoms.

**Supplementary Table 3. Factors associated with the risk of symptom occurrence in COVID-19 infected individuals: Multivariate Logistic regression analysis, N=2,066/983.**

| **Variables** | **First infection, N=2,066** | | | |  | **Second infection, N=983** | | | |
| --- | --- | --- | --- | --- | --- | --- | --- | --- | --- |
|  | **Prevalence of any symptom^a^** | **Risk of any symptom occurrence**  **(Multivariate Logistic regression analysis)^b^** | | |  | **Prevalence of any symptom^a^** | **Risk of any symptom occurrence**  **(Multivariate Logistic regression analysis)^b^** | | |
|  | **%** | **Adjusted OR** | **95% CI** | ***P-value*** |  | **%** | **Adjusted OR** | **95% CI** | ***P-value*** |
| **Total** | 81.4 | - | - | - |  | 55.5 | - | - | - |
| **Timing of the first COVID-19 infection** |  |  |  |  |  |  |  |  |  |
| Before Feb 2023 (BA.5.2/BF.7 predominance period) | 89.6 | 1.00 | - | - |  | 56.6 | 1.00 | - | - |
| After March 2023 (XBB.1.9.1/XBB.1.16/EG.5.1/JN.1 predominance period) | 45.2 | **0.09** | **0.06-0.13** | **<0.001** |  | 39.1 | 0.42 | 0.15-1.18 | 0.101 |
| **Presence of any symptoms during the first infection^a^** |  |  |  |  |  |  |  |  |  |
| No | - | - | - | - |  | 40.4 | 1.00 | - | - |
| Yes | - | - | - | - |  | 57.8 | **2.01** | **1.27-3.16** | **0.003** |
| **Gender** |  |  |  |  |  |  |  |  |  |
| Male | 79.2 | 1.00 | - | - |  | 53.7 | 1.00 | - | - |
| Female | 83.0 | 1.15 | 0.86-1.52 | 0.344 |  | 56.9 | 1.17 | 0.89-1.54 | 0.250 |
| **Age (years)** |  |  |  |  |  |  |  |  |  |
| <18 | 76.4 | 1.00 | - | - |  | 52.4 | 1.00 | - | - |
| 18-35 | 85.0 | 0.71 | 0.29-1.72 | 0.445 |  | 63.1 | 0.73 | 0.30-1.80 | 0.500 |
| 36-60 | 81.0 | 0.50 | 0.22-1.18 | 0.113 |  | 56.6 | 0.60 | 0.25-1.42 | 0.247 |
| >60 | 81.9 | 0.64 | 0.30-1.37 | 0.251 |  | 38.4 | **0.32** | **0.15-0.72** | **0.006** |
| **Education level** |  |  |  |  |  |  |  |  |  |
| Junior high school or below | 76.7 | 1.00 | - | - |  | 49.1 | 1.00 | - | - |
| Senior high school or vocational school | 79.8 | 0.99 | 0.63-1.55 | 0.962 |  | 53.3 | 1.26 | 0.81-1.97 | 0.313 |
| College, university or above | 84.8 | 1.34 | 0.84-2.14 | 0.217 |  | 59.1 | 1.04 | 0.67-1.63 | 0.864 |
| **Occupational categories** |  |  |  |  |  |  |  |  |  |
| Manual laborers | 81.3 | 1.00 | - | - |  | 54.4 | 1.00 | - | - |
| Non-manual laborers | 84.9 | 1.02 | 0.67-1.54 | 0.934 |  | 63.5 | 1.44 | 1.00-2.06 | 0.049 |
| Unemployed or retired individuals | 78.5 | 0.95 | 0.55-1.64 | 0.851 |  | 44.7 | 0.65 | 0.39-1.08 | 0.097 |
| Others or freelancers | 80.6 | 0.65 | 0.40-1.04 | 0.070 |  | 58.8 | 1.18 | 0.76-1.82 | 0.470 |
| **BMI (kg/m^2^)** | - | **1.06** | **1.01-1.10** | **0.013** |  | - | 0.98 | 0.94-1.02 | 0.229 |
| **Number of doses of COVID-19 vaccine administered** |  |  |  |  |  |  |  |  |  |
| 0 | 82.0 | 1.00 | - | - |  | 55.9 | 1.00 | - | - |
| 1 or 2 | 77.1 | 0.69 | 0.40-1.22 | 0.202 |  | 51.0 | 0.79 | 0.46-1.36 | 0.397 |
| 3 or 4 | 82.6 | 0.93 | 0.63-1.37 | 0.724 |  | 56.5 | 0.91 | 0.64-1.29 | 0.579 |
| **Presence of chronic or other underlying diseases** |  |  |  |  |  |  |  |  |  |
| No | 81.2 | 1.00 | - | - |  | 55.5 | 1.00 | - | - |
| Yes | 82.9 | 1.07 | 0.65-1.76 | 0.800 |  | 56.1 | **1.66** | **1.05-2.62** | **0.031** |

^a^The presence of any symptom among fever, cough/expectoration, fatigue, reduced sense of smell/taste, nasal congestion, runny nose, sore throat, conjunctivitis, or diarrhea was defined as having symptoms. Participants who self-reported symptom duration in the ranges of 1-3 days, 4-7 days, 1-2 weeks, or 2-4 weeks were classified under the category ‘Yes’.

^b^Among participants who contracted COVID-19 once or twice by the time of the last follow-up, the occurrence of any infection symptom was used as the dependent variable. A Logistic regression models was utilized to explore factors associated with the occurrence of symptoms.

**Supplementary Table 4. Factors associated with the risk of Long COVID occurrence in COVID-19 infected individuals: Univariate Logistic regression analysis, N=2,868.**

| **Variables** | **Long COVID (lasting >3 months)^b^** | | | |
| --- | --- | --- | --- | --- |
|  | **Presence of any Long COVID^a^** | **Risk of any Long COVID occurrence**  **(Univariate Logistic regression analysis)^b^** | | |
|  | **%** | **OR** | **95% CI** | ***P-value*** |
| **Total** | 3.8 | - | - | - |
| **Gender** |  |  |  |  |
| Male | 2.3 | 1.00 | - | - |
| Female | 4.9 | **2.17** | **1.40-3.35** | **0.001** |
| **Age (years)** |  |  |  |  |
| <18 | 0.8 | 1.00 | - | - |
| 18-35 | 2.3 | **3.51** | **1.22-10.11** | **0.020** |
| 36-60 | 3.6 | **4.36** | **1.58-12.06** | **0.005** |
| >60 | 3.0 | **3.69** | **1.20-11.32** | **0.022** |
| **Education level** |  |  |  |  |
| Junior high school or below | 2.2 | 1.00 | - | - |
| Senior high school or vocational school | 2.7 | 1.16 | 0.62-2.18 | 0.637 |
| College, university or above | 3.2 | **1.74** | **1.05-2.89** | **0.031** |
| **Occupational categories** |  |  |  |  |
| Manual laborers | 3.3 | 1.00 | - | - |
| Non-manual laborers | 2.7 | 1.00 | 0.63-1.58 | 0.983 |
| Unemployed or retired individuals | 2.2 | 0.65 | 0.38-1.12 | 0.118 |
| Others or freelancers | 3.0 | 0.97 | 0.51-1.86 | 0.935 |
| **BMI (kg/m^2^)** | - | 1.01 | 0.96-1.06 | 0.765 |
| **Number of doses of COVID-19 vaccine administered** |  |  |  |  |
| 0 | 4.0 | 1.00 | - | - |
| 1 or 2 | 1.9 | **0.34** | **0.17-0.67** | **0.002** |
| 3 or 4 | 2.7 | **0.62** | **0.40-0.95** | **0.028** |
| **Presence of chronic or other underlying diseases** |  |  |  |  |
| No | 2.5 | 1.00 | - | - |
| Yes | 5.5 | **2.87** | **1.83-4.51** | **<0.001** |

Abbreviation: OR, Odds ratios; CI, confidence intervals; BMI, body mass index;

^a^The presence of any Long COVID symptom among cough, fatigue, reduced sense of smell/taste, myalgia, diarrhea, dizziness/headache, sleep disturbances, cognitive impairments, mood disorders, chest tightness/breathing difficulties, loss of appetite, rash, or hair loss was defined as having Long COVID symptoms. Each symptom was documented through self-reporting by the participants. Participants who report a duration of ‘lasting >3 months’ were considered to be experiencing that particular Long COVID symptom.

^b^In participants who had previously been infected once or twice at baseline, the occurrence of any Long COVID (lasting >3 months) was used as the dependent variable. A Logistic regression model was employed to investigate factors associated with the risk of Long COVID occurrence.
